# Antimicrobial Resistance and Transconjugants Characteristics of *sul3* Positive *Escherichia coli* Isolated from Animals in Nanning, Guangxi Province

**DOI:** 10.3390/ani12080976

**Published:** 2022-04-10

**Authors:** Qinmei Li, Zheng Li, Yuhan Wang, Yunru Chen, Junying Sun, Yunqiao Yang, Hongbin Si

**Affiliations:** State Key Laboratory for Conservation and Utilization of Subtropical Agro-Bioresources, College of Animal Science and Technology, Guangxi University, Nanning 530004, China; 1818393018@st.gxu.edu.cn (Q.L.); 1718306003@st.gxu.edu.cn (Z.L.); 1918393052@st.gxu.edu.cn (Y.W.); 1818393005@st.gxu.edu.cn (Y.C.); 1818393033@st.gxu.edu.cn (J.S.); yunqiaoyang@st.gxu.edu.cn (Y.Y.)

**Keywords:** *sul3*, multiple drug resistance, antimicrobial resistance gene, plasmid

## Abstract

**Simple Summary:**

*Escherichia coli* (*E. coli*) is a common pathogen able to cause infection in humans and animals, especially in Nanning and other areas with intensive livestock and poultry industry. In order to prevent infection in livestock and poultry, sulfonamides are widely used, which accelerate the emergence and enrichment of sulfonamides resistance genes. This manuscript describes an epidemiological survey of *sul3*-positive pathogenic *E. coli* isolates in Nanning, assessing two vital features: antimicrobial resistance and transconjugants. All *sul3* positive pathogenic *E. coli* were multidrug-resistant bacteria. *Sul3* has the potential to transfer among *E. coli*, coupled with the contact between humans and animals. Under the circumstances, long-term monitoring is helpful to control the prevalence of drug resistance in Nanning.

**Abstract:**

Sulfonamides are the second most popular antibiotic in many countries, which leads to the widespread emergence of sulfonamides resistance. *sul3* is a more recent version of the gene associated with sulfonamide resistance, whose research is relatively little. In order to comprehend the prevalence of *sul3* positive *E. coli* from animals in Nanning, a total of 146 strains of *E. coli* were identified from some farms and pet hospitals from 2015 to 2017. The drug resistance and prevalence of *sul3 E. coli* were analyzed by polymerase chain reaction (PCR) identification, multi-site sequence typing (MLST), drug sensitivity test, and drug resistance gene detection, and then the plasmid containing *sul3* was conjugated with the recipient strain (C600). The effect of *sul3* plasmid on the recipient was analyzed by stability, drug resistance, and competitive test. In this study, forty-six *sul3* positive *E. coli* strains were separated. A total of 12 ST types were observed, and 1 of those was a previously unknown type. The ST350 is the most numerous type. All isolates were multidrug-resistant *E. coli*, with high resistant rates to penicillin, ceftriaxone sodium, streptomycin, tetracycline, ciprofloxacin, gatifloxacin, and chloramphenicol (100%, 73.9%, 82.6%, 100%, 80.4%, 71.7%, and 97.8%, respectively). They had at least three antibiotic resistance genes (ARGs) in addition to *sul3*. The plasmids transferred from three *sul3*-positive isolates to C600, most of which brought seven antimicrobial resistance (AMR) and increased ARGs to C600. The transferred *sul3* gene and the plasmid carrying *sul3* could be stably inherited in the recipient bacteria for at least 20 days. These plasmids had no effect on the growth of the recipient bacteria but greatly reduced the competitiveness of the strain at least 60 times in vitro. In Nanning, these *sul3*-positive *E. coli* had such strong AMR, and the plasmid carrying *sul3* had the ability to transfer multiple resistance genes that long-term monitoring was necessary. Since the transferred plasmid would greatly reduce the competitiveness of the strain in vitro, we could consider limiting the spread of drug-resistant isolates in this respect.

## 1. Introduction

The problem of bacterial resistance has a long history, which has become a medical problem to be reckoned with now. Many resistances in bacteria are dominated by mobile genetic elements, including plasmids, integrons, and transposons [1]. *E. coli* is a common Gram-negative bacteria and one of the symbiotic bacteria in the intestine and environment of most livestock and poultry. However, many studies have also shown that *E. coli* can cause a variety of diseases in humans and animals [2,3]. Antibiotics have been used to treat bacterial infections and even as feed additives to promote the growth of livestock and poultry for a long-term period [1,4]. Used antibiotics are not completely absorbed or metabolized by the organism [5]. After being discharged, these antibiotics can pollute and spread in the environment in a variety of ways, such as agricultural runoff, sewage discharge, and nearby farm leaching [6]. Therefore, many symbiotic bacteria such as *E. coli* have to live in an environment containing antibiotics for a long time, which provides appropriate selection pressure for the emergence and spread of multi-antibiotic-resistant bacteria and antibiotic resistance genes (ARGs).

Sulfonamide is a kind of antibiotic with a low soil adsorption rate and high mobility, which is not easy to degrade [7,8]. It competes for binding sites of the dihydro-pteroate synthase (DHPS) enzyme and p-aminobenzoic acid to inhibit the growth and reproduction of bacteria [9]. Moreover, sulfonamide also has the advantages of extensive use, low cost, and wide variety. Since the first sulfonamide was used clinically in 1935, it has been regarded as one of the commonly used antibiotics in the prevention and treatment of aquatic and livestock diseases [10,11]. 

Sulfonamide resistance (*sul*) genes, including *floP* and *sul*, can encode a kind of DHPS with low affinity to sulfonamides, which makes bacteria grow and reproduce normally in an environment containing sulfonamides [12,13]. At present, four kinds of sulfonamides resistant genes (*sul1, sul2, sul3 and sul4*) have been found in plasmids. *sul1* and *sul2* were discovered successively in 1985 [14,15]. *sul4* was a sulfonamides resistant type recently discovered in Swiss swinery [16], which had also been found in the type I integron transmission gene observed in Indus River sediments, while was not reported in clinical isolates [9]. Martin et al. found a gene similar to *sul1* in Mycobacterium, which had missed the promoter codon, and the codon had been inserted further upstream, so the gene was named *sul3* gene in 1990 [17]. Since its discovery, *sul3* has been successively found in more and more regions, sources, and strains [18,19,20,21], among which even belong to human-originated *E. coli* [22]. 

Nanning is the capital of Guangxi Province, located in southwest China. The breeding industry in Nanning is mainly composed of retail investors. The unreasonable use of antibiotics for livestock and poultry diseases, coupled with the lack of effective management measures, perpetuates the problem of bacterial resistance. The purpose of this study is to detect the antimicrobial resistance, multi-locus sequence typing (MLST), and antimicrobial resistance gene characteristics of *sul3* positive *E. coli* from animals in the Nanning area. At the same time, to evaluate the influence of *sul3* positive bacteria on host bacteria after conjugation. 

## 2. Methods

### 2.1. Sample Collection and Processing

The urban area of Nanning mainly includes Qingxiu District, Xingning District, Jiangnan District, Liangqing District, Yongning District, Xixiangtang District, and Wuming District. These areas are home to more than 90% of the leading breeding enterprises in Nangning. From 2015 to 2017, the farms were selected randomly in Nanning city to ensure representative production, covering commercial type, semi-commercial type, and backyard. Pig farms with pig age ≥20 weeks and poultry farms with poultry age ≥12 weeks were selected. The source range of pet dogs was at least covered in 3 different districts by animal hospitals. Finally, 20 farms and 4 animal hospitals were determined and enrolled in this study. For selected poultry farms and pig farms, 5% of the age-appropriate number were identified for sampling. As for selected animal hospitals, the sampling quantity was carried out in accordance with the proportion of 20%. A total of 150 fecal samples were collected in Nanning, and thereinto, 59 samples from 12 chicken farms, 38 samples from 8 pig farms, and 53 samples from 4 animal hospitals. All samples were stored in sterile EP tubes at 4 °C and then transported to the Clinical Veterinary Laboratory of Guangxi University within four hours for immediate processing upon receipt.

### 2.2. Isolation and Identification of sul3 Positive E. coli

The fecal samples were cultured in 3.5 mL LB broth (AOBOX, Beijing, China) at 37 °C in a constant temperature shaking shaker for 8 h. Bacteria were streaked into McConkey agar (Huankai, Guangdong, China) plate by sterile inoculation ring and incubated in a constant temperature incubator at 37 °C for 16–18 h. A single rosy round smooth colony was selected from McConkey agar (Huankai, Guangdong, China) plate, and the above steps were repeated for repeated purification. The purified strains were inoculated on Eosin methylene blue agar (Huankai, Guangdong, China) plate and cultured in a constant temperature incubator at 37 °C for 18–24 h. A single suspected *E. coli* strain is selected, whose appearance is characterized by a smooth round colony with black and green metallic luster in the center. The DNA of bacteria was extracted by the boiling method. *E. coli* was shaken, culturing at 37 °C for 8 h, 1.5 mL bacterial liquid was taken and absorbed into an EP tube, centrifuged at 14,000 rpm for 2 min, and the supernatant was discarded. After the thallus was obtained, sterile distilled water was added, mixed, and placed in boiling water for 15 min, followed by an ice bath for 5 min, centrifuged at 14,000 rpm for 2 min, and the supernatant could be taken. The universal primer designed by Wu Yongji [23] and *sul3* primer reported by Wang Yayun [24] were respectively sent to Shanghai Sangon Bioengineering Co., Ltd. (Shanghai, China) for synthesis (Table 1). The above-extracted DNA was used as the template for Polymerase chain reaction (PCR) amplification. The total PCR reaction system was 25 μL: 1 μL forward primer (Sangon Biotech, Shanghai, China), 1 μL reverse primer (Sangon Biotech, Shanghai, China), 2 μL template, 12.5 μL mix (GenStar, Beijing, China) and 8.5 μL deionized water (Sangon Biotech, Shanghai, China). PCR reaction procedure: pre-denaturation at 94 °C for 5 min. A total of 30 cycles included denaturation at 94 °C for 30 s, annealing at 55 °C for 30 s (depending on different primers), extension of 1 min at 72 °C, and then extension of 10min at 72 °C. The PCR products were sent to the company for sequencing and uploaded to National Center for Biotechnology Information (NCBI) for Basic Local Alignment Search Tool (BLAST) confirmation of suspected isolates and *sul3* carrier. Determine whether the strain is *E. coli* by the results of 16Sr RNA sequencing. *sul3* carrier was used to determine whether these *E. coli* carried *sul3* gene, and the confirmed *sul3* positive *E. coli* was named E1-E46. These *sul3* positive *E. coli* were preserved with 30% glycerol (*v/w*), and the preserved isolates and their extracted DNA samples were stored in different refrigerators at −20 °C for follow-up study.

### 2.3. MLST Typing Detection

A total of 46 strains of *sul3* positive *E. coli* were detected. PCR amplification was conducted using 7 pairs of primers (*adk, fumC, gyrB, icd, mdh, purA and recA*) (Table 1 and Table S1). The reaction system and conditions are consistent with described earlier. A total of 46 strains were typed by MLST, and the positive products were sent to Wuhan Jinkairui Biological Engineering Co., Ltd. (Wuhan, China) for one-way sequencing, and the results were submitted to the MLST website (https://pubmlst.org/escherichia/) (accessed on 18 November 2019) for further testing. After obtaining the allele factor spectrum, the ST type was checked on the website (http://enterobase.warwick.ac.uk/species/ecoli/allele_st_search) (accessed on 18 November 2019).

### 2.4. Antibiotic Sensitivity Experiment

The MIC of antimicrobial agents against *sul3* positive *E. coli* was used by the broth dilution method recommended, which was recommended by 2017 Clinical and Laboratory Standards Institute (CLSI). The concentration of *E. coli* was prepared into 10^5^ CFU/mL. The tested antimicrobial agents included penicillin, ceftazidime, ceftriaxone, meropenem, amikacin, streptomycin, tetracycline, ciprofloxacin, gatifloxacin, chloramphenicol, fosfomycin, and colistin. The results of antibiotic sensitivity were also judged according to the break-point standard established by 2017 CLSI (Table 2). The *E. coli* of ATCC 25922 was used for the quality control of antibiotic sensitivity test. According to the method reported by Ibrahim YK, multiple antibiotic resistance indices (MARI) of 46 *sul3* positive strains were assessed [36]. Similarly, the concentration of transconjugants was prepared into 10^5^ CFU/mL. Seven antibiotics include penicillin, ceftazidime, streptomycin, amikacin, tetracycline, ciprofloxacin, and chloramphenicol. The rest are consistent with the foregoing. The receptor bacteria (C600) and the donor bacteria (EC027,EC035,EC038) were used as reference to judge the drug resistance of the three conjugates.

### 2.5. Resistant Genes Detection

Using previously extracted DNA as template, 24 antibacterial genes were detected, including the β-lactam (*bla*_TEM_, *bla*_CTX-M9_*,* *bla*_CTX-MU_ and *bla*_OXA-1_*)*, aminoglycosides (*armA, rmtA, rmtB, aac(6’)–Ib* and *aac(3’)-II*), tetracyclines (*tetA, tetB* and *tetM*), quinolones (*qnrA* and *qnrB*), sulfonamides (*sul1* and *sul2*), and other classes (*floR, mcr-1, oqxA, oqxB*, and *fosA3*) (Table 1). The PCR program is consistent with the previous description.

### 2.6. Conjugative Experiment

The conjugative experiment was conducted by filter membrane method. The *E. coli* C600, which did not produce acid and has rifampicin resistance, was used as the recipient bacteria. *sul3*-positive isolates were used as the donor bacteria. The donor and recipient bacteria were mixed with 0.5 Mcfarland concentration at 1:4 and added to an Agar plate affixed with a filter membrane, and cultured overnight at 37 °C. The filter membrane was put into the broth to dissolve the attached bacteria. The transconjugants were screened from McConkey medium with a concentration of 6000 μg/L sulfamethazine and 3500 μg/L rifampicin. The suspected transconjugants were subjected to PCR and antibiotics sensitivity tests to confirm whether the plasmid transfer carried *sul3* was successful, and then enterobacterial repetitive intergenic consensus (ERIC)-PCR was used to determine the correlation between the transconjugants and C600, with the ERIC-primers as described previously [28] (Table 1). Combined with antibiotics sensitivity tests and drug resistance gene test results, we can know whether there are other resistance genes co-transferred with *sul3.*

### 2.7. Growth Curve

We used absorbance method to observe the change in the growth status of transconjugants and C600, specifically as follows. After shaking culture at 37 °C overnight, the bacterial solution was added to fresh LB broth according to the ratio of 1:1000. For a total of 16 time points, 3 mL was taken from each time point for 600 optical density (OD_600_). The observation lasted for 24 h and needed to be repeated 3 times in parallel. 

### 2.8. In Vitro Competitive Test

The competitive experiment was conducted with previous descriptions [37] to compare the nutritional competitiveness of transconjugants with recipient bacteria without *sul3* plasmids in vitro. According to the drug sensitivity test of transconjugants, the tested antibacterial agent was streptomycin. First, two kinds of bacteria were cultured to 0.5 McFarland concentration, then mixed according to the proportion of 1:1, added to 10mL LB broth, incubated at 37 °C and 220 r/min for 16 h. After being diluted 10^6^ times, 100 μL bacterial solution was respectively coated with 60 μg/mL streptomycin LB agar and streptomycin-free LB agar, and cultured overnight at 37 °C. The total colony-forming unit (CFU) and streptomycin-resistant CFU were counted, and the competition index of non-resistant CFU and streptomycin-resistant CFU was calculated. The parallel experiment was repeated 3 times.

### 2.9. Plasmid Stability

According to the previous description of plasmid stability [38], the transconjugants were shaken in LB medium at 37 °C, 220 rmp for 12 h, and regarded as the first generation of transconjugants. Then the first generation transconjugants were inoculated in new LB medium and shaken at 37 °C for 12 h again, repeated every 12 h. Each time was counted as one generation, and the procedure was repeated for 60 generations. Every 10 generations, part of the bacterial solution was diluted and coated with agar medium, 24 colonies of bacteria were randomly selected to extract DNA by boiling method, and then *sul3* PCR was carried out to determine the positive rate of *sul3*.

### 2.10. Statistical Analysis

Results are shown as mean ± SD; statistical significance is indicated as follows: *p < 0.05, and NS means no significance. GraphPad Prism 6.01 software (GraphPad Software Inc., San Diego, CA, USA) was used for analysis via one-way analysis of variance (One-way ANOVA).

## 3. Results

### 3.1. Isolates and MLST

From 2015 to 2017, 142 strains of *E. coli* were detected from 150 samples of animal origin in Nanning, among which 46 strains carried *sul3*, accounting for 32.4% of the total number of *E. coli* isolates. The 46 strains of *sul3* positive *E. coli* were divided into 12 ST genotypes in total. Overall, ST746 was the dominant cluster (13, 28.2%); both it and ST156 were identified in chickens. ST10, ST746 and ST641 were detected among isolates from chickens (*n* = 2, 2, 1) and pigs (*n* = 2, 3, 3). ST101 was identified in pigs (*n* = 2). ST2178 strains were detected in isolates of dogs (*n* = 4) and pigs (*n* = 2). Finally, the sample of the unknown type is from a pig (Table 3).

### 3.2. Antibiotic Resistance and Resistance Gene 

The results showed that 46 strains of *sul3* positive *E. coli* were highly resistant to penicillin, ceftriaxone, streptomycin, tetracycline, ciprofloxacin, gatifloxacin, and chloramphenicol, which were 100% (46/46), 73.9% (34/46), 82.6% (38/46), 100% (46/46), 80.4% (37/46), 71.7% (33/46) and 97.8% (45/46), Some strains were also resistant to amikacin and colistin (10.9%, 5/46), only sensitive to meropenem (Table 4). All *sul3* positive *E. coli* had MARI > 0.2; that is to say, they are all multi-resistant bacteria. In addition to *sul3*, 20 kinds of antimicrobial resistance genes were detected, of which *tetA* (95.7%, 44 / 46), *floR* (89.1%, 41 / 46), *oqxA* (76.1%, 35 / 46), *sul2* (80.4%, 37 / 46) were detected of rate higher, and strains carrying *mcr-1* (21.7%, 10 / 46) were also detected, *armA* and *bla*_SHV_ was not detected (Table 3 and Table 5). 

### 3.3. Transconjugants and Related Experiments

**Figure 1:**
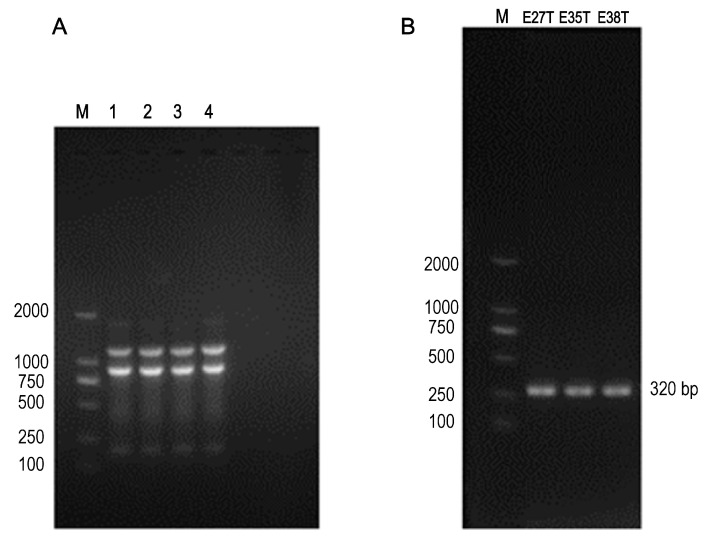
The ERIC-PCR and PCR results. (**A**) Lanes 1–3: transconjugants EC027/T, EC035/T and EC038/T, lanes 4: C600, M: 2000 DNA marker; The ERIC-PCR result of 3 transconjugants and C600, indicating that these transconjugants and C600 were homologous strains. (**B**) E27T: EC027/T; E35T: EC035/T; E38T: EC038/T; *Sul3* gene was detected in the above three transconjugants.

Three suspected transconjugants were successfully obtained through the conjugation experiment. After *sul3* positive identification and ERIC-PCR (Figure 1A), the three suspected transconjugants were all the plasmid strains obtained from the recipient bacteria (C600), named EC027/T, EC035/T, and EC038/T according to the donor bacteria name (Figure 1B). 

**Figure 2:**
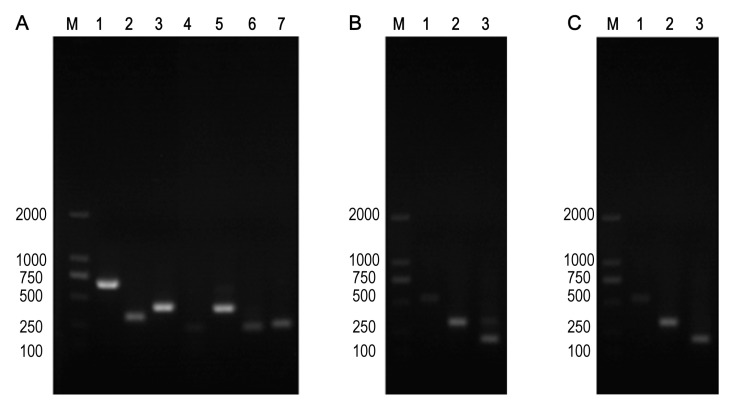
Three transconjugants strains contained drug resistance genes. (**A**) EC027/T, Note: M: 2000 DNA Marker; Lanes 1–7: *bla*_OXA-1_*, sul3, tetM, floR, aac(6’)-Ib, sul2, sul1*. (**B**) EC035/T, note: M: 2000 DNA Marker; Lanes 1–3: *bla*_TEM_*, sul3, tetA*. (**C**) EC038/T, note: M: 2000 DNA Marker; Lanes 1–3: *bla*_TEM_*, sul3, tetA*.

**Figure 3:**
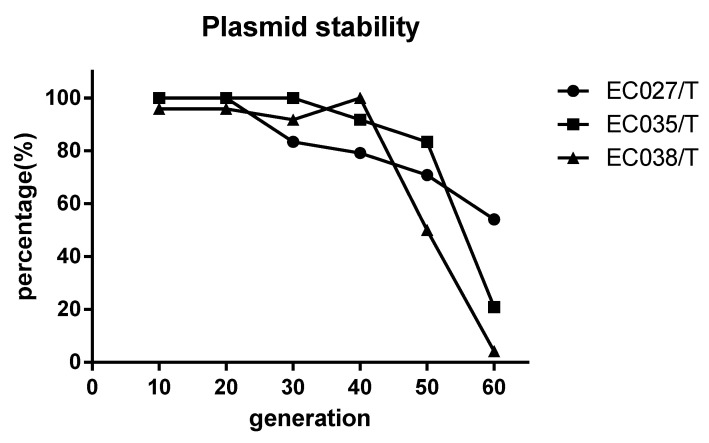
Stability test of *sul3* positive wild plasmid. The positive rate of *sul3* remained above 70% when the transconjugants were passed on to the 20th day (40 generations), indicating that the *sul3* plasmid could be inherited stably for a long time in the transconjugants.

In comparison with the recipient bacteria, the Minimum Inhibitory Concentration (MIC) of the maximum seven antimicrobials in transconjugants (EC027/T) showed different degrees of elevation, including penicillin, ceftazidime, streptomycin, amikacin, tetracycline, ciprofloxacin and chloramphenicol (Table 6). According to the detection results of resistance genes, in addition to the *sul3* gene, E027/T was detected with six new resistance genes, while E025/T and E038/T were two (Table 7, Figure 2). However, referring to the sensitivity of the transconjugants to antibacterial drugs (Table 6), we verified that no corresponding resistance genes of streptomycin and chloramphenicol were detected via PCR (Table 7). The plasmid stability experiment showed that the plasmid could be stable and continuously passed for at least 40 generations; that is to say, it had strong stability in 20 days (Figure 3). 

### 3.4. The Adaptive Cost of Plasmid C600

**Figure 4:**
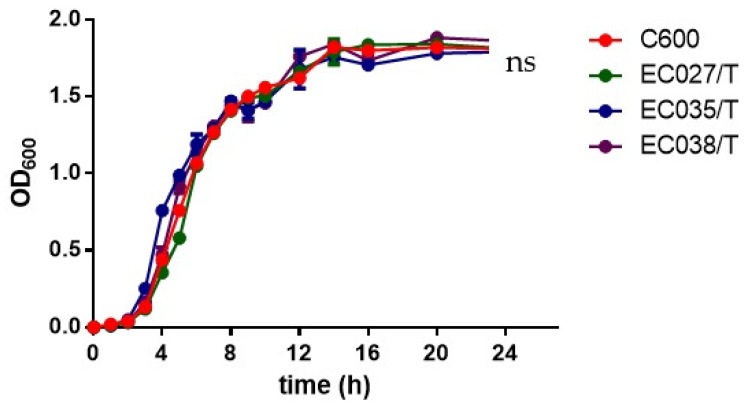
Growth curves for 3 transconjugants and C600. There was no overall significant difference between the growth curve of zygons and the growth curve of C600 (red) (*p* > 0.05).

**Figure 5:**
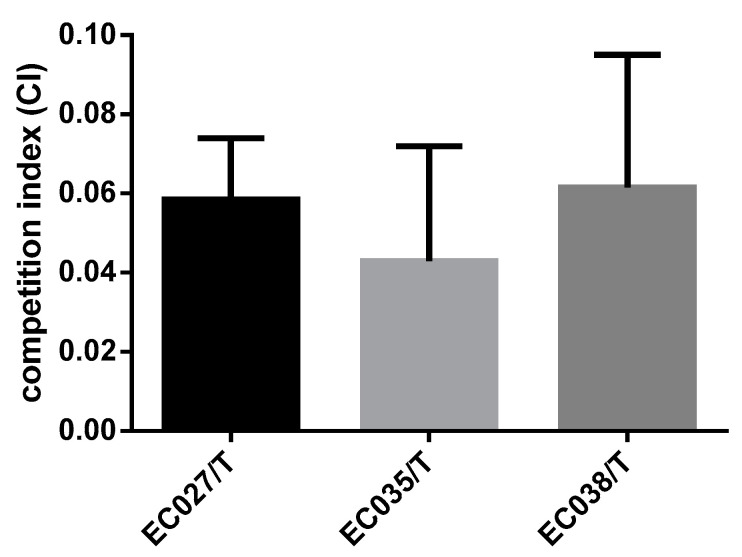
The competitive index of extracorporeal competition.

The growth curves of the three transconjugants and the recipient bacteria showed that the transconjugants and the recipient bacteria had minor changes only during the logarithmic growth period, and the changes were not obvious after entering the stable period at 8 h (*p* *>* 0.05) (Figure 4). It indicates that the transconjugants have little influence on the growth of the recipient bacteria. In the competitive test, we observed that the competitive index of the three transconjugants was significantly reduced compared with that of the recipient bacteria C600, among which the most obvious one was EC035/T (0.043), followed by EC027/T (0.058) and EC038/T (0.061) (Figure 5). The competition index indicated that the ratios between the CFU of the streptomycin-resistant strain and the streptomycin-sensitive strain were all less than 0.08, revealing that the competitive ability of the transconjugants was greatly weakened in vitro.

## 4. Discussion

Despite the fact that sulphonamides are rarely used to treat human bacterial infections in many regions, they are still widely used in aquaculture, animal husbandry, and veterinary practice because of the lower price [20]. Sulfonamides can penetrate into rivers and water sources through soil, and the detection of its concentration is a priority indicator to judge the effectiveness of sewage treatment. Massive use plus great potential for penetrating into the environment leads to the extensive spread of *sul* genes. In this study, 46 strains carrying *sul3* genes were screened from 142 *E. coli*, and the detection rate was 32.4%. Several studies in recent years showed that the detection rate of *sul1* and *sul2* in sulfonamides-resistant genes was higher than that of *sul3* [39,40,41,42], which hinted that the situation of sulfonamides resistance in the Nanning area might be more serious and needed to be paid close attention to.

We reported the prevalence of *sul3*-positive *E. coli* in Nanning for the first time. To further understand the typing of *sul3*-positive *E. coli* in Nanning, we carried out MLST detection. In the test, the diversity of each *sul3* positive strain was low, but there are still more common types of ST typing. ST23, ST156, and ST10 were reported to be related to humans [43,44,45]. Among them, ST10 is the most common pedigree in human urine *E. coli* isolates [43], and these reports also pointed out that these three types were also found in other *E. coli* strains. Although these three types were rarely detected in this study, it is still necessary to pay attention to the transmission between humans and livestock.

The emergence of multidrug-resistant bacteria seriously affects the cure rate of bacterial infection diseases, becoming a potential threat to the health of human beings and livestock [46]. In this study, it was found that all *sul3*-positive strains were multiple AMR bacteria with at least three multiple drug resistance and carried at least six drug resistance genes simultaneously through antibiotic sensitivity experiment and partial ARGs detection. Interestingly enough, we found *sul2* was present in 80.4% of the 46 *sul3* positive isolates, and *sul1* accounted for 30.4%. The base sequences of *sul1*, *sul2,* and *sul3* are about 50% homologous to each other [47]. *Sul2* genes are located on large multi-resistance plasmids with a broad host range and are more common in clinics [14,48,49]. It might explain the high proportion of *sul2* gene in 46 sul3-positive strains. Although the data in this study supported the close correlation between *sul2* and *sul3*, further direct evidence was needed to prove the synergistic effect of two genes on antibiotic resistance. Among the tested antibiotics, only meropenem was completely sensitive, and AMR was serious, which verified our previous conjecture. Plasmids are circular DNA double strands in bacteria, which can be transcribed and expressed independently of bacterial nucleic acids, and are regarded as the main way for the rapid spread of drug resistance. In the conjugative experiment, we detected the AMR and ARGs of the conjugates. It was worth noting that only quinolones and aminoglycosides had differences in the detection rate of resistance genes and AMR rate in isolated strains. Similarly, no ARGs associated with streptomycin and chloramphenicol were detected in conjugates. It suggested that there might be other related genes mediating the tolerance of the above-mentioned antimicrobials, which might be the efflux pump or the resistance genes of the relevant antimicrobials. Regarding the plasmids in these isolates, we could not determine the type and quantity of these transfer plasmids. What we could confirm was that after acquiring the plasmid, there were several (at least 4) antibiotics resistance changes to the strains corresponding to the tested resistance genes. It indicated that the transferred ARGs could be expressed via host cells, which might affect the effective use of antibiotics in Nanning.

After analyzing the genetic environment of the *sul* gene, Jang [20] concluded that compared with the other two genes, the diversity of adjacent genetic transfer elements and the *sul3* resistance genes were lower, and some *sul3* even existed on chromosomes, which affected the transmission of *sul3*. However, some studies manifested that *sul3* is related to type I integron and could replace *sul1* to form an atypical type I integron [20,50]. In addition, heavy metals in the environment were also beneficial to the spread of *sul3* [51,52]. Our research also indirectly reflected the potential of *sul3* to spread widely. The stability test showed that the transferred *sul3* wild plasmid could be inherited in bacteria for a long time. The growth curve showed that the *sul3* plasmid had no effect on the growth performance of the strain, which was consistent with the previous report [36,45,53]. Fortunately, the wild plasmid in this study reduced the competitiveness of the host bacteria in vitro by at least 60 times. Although the types and quantities of drug-resistant genes studied were different, this also indicated that wild plasmids would bring a greater adaptive cost to the recipient bacteria due to multiple drug-resistant genes or other unknown genes.

## 5. Conclusions

Forty-six *sul3* positive strains of *E. coli* carried multiple-drug resistance genes and have serious AMR. *sul3* wild plasmid could transmit a variety of ARGs, enhance the resistance of bacterial receptors to antibiotics, and pose a potential threat for antibiotic use in Nanning in the future. However, wild plasmid *sul3* could also reduce the competitiveness of strains in vitro, which is also a breakthrough in prevention and treatment.

## Figures and Tables

**Table 1:** PCR Primers.

Gene	Primer Sequence (5′→3′)	Product Size (bp)	Annealing Temp (°C)	References
*16Sr RNA*	Fw: AGAGTTTGATCCTGGCTCAG	1466	55	[23]
	Rev: ACGGCTACCTTGTTACGACTT			
*bla*_TEM_	Fw: AGGAAGAGTATGATTCAACA	511	52.5	[23]
	Rev: CTCGTCGTTTGGTATGGC			
*bla*_SHV_	Fw: GGTTATGCGTTATATTCGCCTGTG	1031	56.5	[23]
	Rev: TTAGCGTTGCCAGTGCTCGATCA			
*bla*_CTX-M1_	Fw: GGTTAAAAAATCACTGCGTC	864	56	[25]
	Rev: TTGGTGACGATTTTAGCCGC			
*bla*_CTX-M9_	Fw: ATGGTGACAAAGAGAGTGCA	870	50	[26]
	Rev: CCCTTCGGCGATGATTCTC			
*bla*_CTX-MU_	Fw: ATGTGCAGTACCAGTAAAGT	593	56	[24]
	Rev: TGGGTRAAGTARGTCACCAGAA			
*bla*_OXA-1_	Fw: TTGAAGGAACTGAAGGTTGT	651	54	[27]
	Rev: CCAAGTTTCCTGTAAGTGCG			
*armA*	Fw: AGGTTGTTTCCATTTCTGAG	591	55	[28]
	Rev: TCTCTTCCATTCCCTTCTCC			
*rmtA*	Fw: CTAGCGTCCATCCTTTCCTC	635	60	[29]
	Rev: TTTGCTTCCATGCCCTTGCC			
*rmtB*	Fw: ATCAACGATGCCCTCACCTCC	631	61	[28]
	Rev: TTCCACGCCCGCCTAAACT			
*aac(6′)-Ib*	Fw: CAAGAGTCCGTCACTCCATACA	396	61	[30]
	Rev: ATGGAAGGGTTAGGCATCACT			
*aac(3′)-II*	Fw: ACTGTGATGGGATACGCGTC	237	60	[31]
	Rev: CTCCGTCAGCGTTTCAGCTA			
*tetA*	Fw: GCTACATCCTGCTTGCCTTC	210	60	[32]
	Rev: CATAGATCGCCGTGAAGAGG			
*tetB*	Fw: TTGGTTAGGGGCAAGTTTTG	659	65	[32]
	Rev: GTAATGGGCCAATAACACCG			
*tetM*	Fw: GTGGACAAAGGTACAACGAG	406	55	[32]
	Rev: CGGTAAAGTTCGTCACACAC			
*qnrA*	Fw: CAAGAGGATTTCTCACGCCAG	628	67	[27]
	Rev: AATCCGGCAGCACTATTACTCC			
*qnrB*	Fw: ATGACGCCATTACTGTATAA	562	57	[27]
	Rev: GATCGCAATGTGTGAAGTTT			
*floR*	Fw: GTCATTCCTCACCTTCATCCTAC	243	60	[31]
	Rev: GACACCAGCACTGCCATTG			
*mcr-1*	Fw: ATGATGCAGCATACTTCTGTG	1626	65	[33]
	Rev: TCAGCGGATGAATGCGGTG			
*oqxA*	Fw: GATCAGTCAGTGGGATAGTTT	670	56	[24]
	Rev: TACTCGGCGTTAACTGATTA			
*oqxB*	Fw: TTCTCCCCCGGCGGGAAGTAC	512	68	[24]
	Rev: CTCGGCCATTTTGGCGCGTA			
*sul1*	Fw: GGCTGGTGGTTATGCACTCA	263	64	[34]
	Rev: CGAGACCAATAGCGGAAGC			
*sul2*	Fw: ACGCAAGCCTATGCCTTGTCG	234	62	[34]
	Rev: TTGCGTTTGATACCGGCACCC			
*sul3*	Fw: CGTAAATATAACCACCGAT	326	55	[34]
	Rev: CCAAGCCTGAATAAATCTCA			
*fosA3*	Fw: GCGTCAAGCCTGGCATTTT	258	55	[23]
	Rev: GCCGTCAGGGTCGAGAAA			
*ERIC-2*	AAGTAAGTGACTGGGGTGACGC	Variable	50	[35]
*adk*	Fw: CTCGCCATTAACCGTTTCAG	739	55	[24]
	Rev: CCAGATCAGCGCGAACTTCA			
*fumC*	Fw: TCACAGGTCGCCAGCGCTTC	769	64	[24]
	Rev: TCCCGGCAGATAAGCTGTGG			
*gyrB*	Fw: ATCGGCGACACGGATGAC	816	66	[24]
	Rev: GTCCATGTAGGCGTTCAGG			
*lcd*	Fw: CCGGCACAAGGCAAGAAGATC	857	59.5	[24]
	Rev: GGACGCAGCAGGATCTGTT			
*mdh*	Fw:GCCTTCAGGTTCAGAACTCTCTCT	798	55	[24]
	Rev: TTCTGTTCAAATGCGCTCAGG			
*purA*	Fw: CGCGCTGATGAAAGAGATGA	817	66	[24]
	Rev: CATACGGTAAGCCACGCAGA			
*recA*	Fw: CGCATTCGCTTTACCCTGACC	731	55	[24]
	Rev:GTCGAAATCTACGGACCGAAT			

**Table 2:** Judgment table of resistance break point of tested antibacterial agents.

Antibiotic Type	Antibiotic Name	Concentration (μg/mL)	CLSI (μg/mL)		
			S	I	R
Beta-lactams	penicillin	5120	≤8	16	≥32
	ceftazidime	6400	≤4	8	≥16
	ceftriaxone	6400	≤1	2	≥4
	meropenem	5120	≤1	2	≥4
Aminoglycosides	streptomycin	6400	≤16	32	≥64
	amikacin	5120	≤16	32	≥64
Tetracyclines	tetracycline	5120	≤4	8	≥16
Quinolones	ciprofloxacin	5120	≤1	2	≥4
	gatifloxacin	6400	≤2	4	≥8
Phenicols	chloramphenicol	5120	≤8	16	≥32
Fosfomycin	fosfomycin	5120	≤64	128	≥256
Polypeptides	colistin	1280	≤2	—	≥4

CLSI: Clinical and Laboratory Standards Institute

**Table 3:** Strain information, MLST typing and antimicrobial resistance gene.

Isolates	Year	Source	ST Type	Antibiotic Resistance Genes (Except for *sul3*)
EC001	2017	pig	641	*tetA-tetM-bla*_TEM_ *-floR-oqxA*
EC004			2178	*tetA-bla* *_CTX-MU_* *-bla*_CTX-M9_ *-floR-mcr-1-sul2-fosA3-oqxA*
EC006			unknown	*aac(3)-II-tetA-sul2*
EC009			222	*tetA-bla*_CTX-MU_ *-bla*_CTX-M9_ *-mcr-1-sul2-fosA3*
EC012			2178	*aac(3)-II-tetA-tetM-bla*_TEM_ *-mcr-1-oqxA-oqxB-sul1-sul2*
EC025			746	*tetA-bla*_TEM_ *-floR-oqxA-sul1-sul2-mcr-1*
EC026			10	*aac(6’)-Ib-tetA-floR-mcr-1-oqxA-sul1-sul2*
EC029			641	*rmtA-tetA-tetM-bla*_TEM_ *-floR-oqxA*
EC038			746	*tetA-bla*_TEM_ *-floR-oqxA-sul1-sul2*
EC041			10	*aac(6’)-Ib-tetA-bla*_CTX-MU_ *-floR-oqxA-oqxB-sul2*
EC022		chicken	350	*tetA-tetM-bla*_TEM_ *-bla*_CTX-MU_ *-bla*_CTX-M9_ *-qnrB-floR-oqxA-sul2*
EC027			156	*aac(6’)-Ib-aac(3)-II-tetA-tetM-bla*_TEM_ *-bla*_CTX-MU_ *-bla*_OXA-1_ *-floR-oqxA-sul1-sul2*
EC028			10	*rmtA-aac(6’)-Ib-tetA-floR-mcr-1-oqxA-sul2*
EC044			457	*aac(3)-II-tetA-tetM-floR*
EC042		dog	2178	*tetA-bla*_CTX-MU_ *-bla*_CTX-M9_ *-floR-oqxA-sul2-fosA3*
EC043			2178	*tetA-bla*_CTX-MU_ *-bla*_CTX-M9_ *-mcr-1-sul2-fosA3*
EC014	2016	pig	101	*tetA-bla*_TEM_ *-bla*_CTX-MU_ *-floR-oqxA-oqxB-sul2-fosA3*
EC034			641	*tetA-bla*_TEM_ *-floR-oqxA-sul1*
EC039			746	*rmtA-tetA-bla*_TEM_ *-oqxA-floR-sul2*
EC003		chicken	350	*tetA-tetM- bla*_TEM_ *-bla*_CTX-MU_ *-bla*_CTX-M9_ *-qnrB-floR-oqxA-sul2*
EC005			350	*tetA-tetM-bla*_CTX-MU_ *-bla*_CTX-M9_ *-floR*
EC013			350	*tetA-tetM-bla*_CTX-MU_ *-bla*_CTX-M9_ *-floR*
EC016			350	*tetA-tetM-bla*_TEM_ *-bla*_CTX-MU_ *-bla*_CTX-M9_ *-qnrB-floR-oqxA-sul1-sul2*
EC017			156	*rmtB-aac(6’)-Ib-aac(3)-II-tetA-tetM-bla*_TEM_ *-bla*_CTX-MU_ *-bla*_OXA-1_ *-floR-oqxA-oqxB-sul1-sul2*
EC018			350	*tetA-tetM-bla*_TEM_ *-bla*_CTX-MU_ *-bla*_CTX-M9_ *-qnrB-floR-sul2*
EC019			350	*tetA-tetM-bla*_TEM_ *-bla*_CTX-MU_ *-bla*_CTX-M9_ *-floR-oqxA-sul2*
EC023			350	*tetA-tetM- bla*_TEM_ *-bla*_CTX-MU_ *-bla*_CTX-M9_ *-qnrB-floR-oqxA-sul2*
EC035			746	*tetA-bla*_TEM_ *-bla*_CTX-MU_ *-floR-oqxA-sul2*
EC036			350	*tetA-tetM- bla*_CTX-MU_ *-bla*_CTX-M9_ *-qnrB-floR-oqxA-sul1-sul2*
EC037			350	*tetA-tetM-bla*_CTX-M9_ *-qnrB-floR-oqxA-sul1-sul2*
EC007		dog	950	*tetA-tetM-bla*_TEM_ *-bla*_CTX-MU_ *-bla*_CTX-M9_ *-floR-oqxA*
EC010			2178	*tetA-bla*_TEM_ *-bla*_CTX-MU_ *- bla*_CTX-M9_ *-floR-mcr-1-oqxA-sul1-sul2-fosA3*
EC011			457	*aac(3)-II-tetA-tetM-bla*_TEM_ *-qnrB-floR-sul2*
EC021			457	*aac(3)-II-tetA-tetM-bla*_TEM_ *-qnrB-floR-oqxA-sul2*
EC040			950	*tetA-tetM-bla*_TEM_ *-bla*_CTX-MU_ *-bla*_CTX-M9_ *-qnrB-floR-oqxA-sul2*
EC031	2015	pig	101	*rmtB-tetA-bla*_TEM_ *-qnrA-oqxA-sul2*
EC002		chicken	457	*aac(3)-II-tetA-tetM-bla*_TEM_ *-qnrB-floR-oqxA-sul2*
EC008			641	*tetA-bla*_TEM_ *-floR*
EC020			457	*aac(3)-II-tetA-tetM-bla*_TEM_ *-qnrB-floR-oqxA-sul2-marA*
EC024			350	*tetA-tetM- bla*_TEM_ *- bla*_CTX-MU_ *- bla*_CTX-M9_ *-qnrB-floR-oqxA-sul2*
EC030			350	*tetA-tetM- bla*_TEM_ *- bla*_CTX-MU_ *-bla*_CTX-M9_ *-qnrB-floR-oqxA-sul1-sul2*
EC032			350	*aac(6’)-Ib-tetA-tetM- bla*_TEM_ *-bla*_CTX-MU_ *-bla*_CTX-M9_ *-qnrB-floR-oqxA-sul1-sul2*
EC033			746	*aac(3)-II-tetB-bla*_TEM_ *-bla*_CTX-MU_ *- bla*_CTX-M9_ *-bla*_OXA-1_ *-floR-sul2*
EC045			10	*tetM-bla*_TEM_ *-bla*_CTX-MU_ *- bla*_CTX-M9_ *-floR-oqxA-sul2-fosA3*
EC046			23	*aac(6’)-Ib-tetA-tetM-bla*_CTX-MU_ *-bla*_CTX-M9_ *-bla*_OXA-1_ *-floR-mcr-1-oqxA-oqxB-sul1-fosA3*
EC015		dog	2178	*tetA-bla*_CTX-MU_ *-bla*_CTX-M9_ *-floR-mcr-1-sul2-fosA3*

**Table 4:** Antimicrobial resistance of *sul3* positive *E. coli*.

Antimicrobial Agents	The Proportion (%) (Positive Number/Total)		
	R	I	S
penicillin	100 (46/46)	0 (0/46)	0 (0/46)
ceftazidime	26.1 (12/46)	13.0 (6/46)	60.9 (28/46)
ceftriaxone	73.9 (34/46)	2.2 (1/46)	23.9 (11/46)
meropenem	0 (0/46)	0 (0/46)	100 (46/46)
amikacin	10.9 (5/46)	0 (0/46)	89.1 (41/46)
streptomycin	82.6 (38/46)	13.0 (6/46)	4.4 (2/46)
tetracycline	100 (46/46)	0 (0/46)	0 (0/46)
ciprofloxacin	80.4 (37/46)	0 (0/46)	19.6 (9/46)
gatifloxacin	71.7 (33/46)	17.4 (8/46)	10.9 (5/46)
chloramphenicol	97.8 (45/46)	2.2 (1/46)	0 (0/46)
fosfomycin	21.7 (10/46)	0 (0/46)	78.3 (36/46)
colistin	10.9 (5/46)	8.7 (4/46)	80.4 (37/46)

Note: R: drug-resistant; I: Degree between resistance and sensitivity; S: sensitive.

**Table 5:** Prevalence of antimicrobia-resistant genes in *sul3* positive *E. coli*.

Drug-Resistant Genes	Positive Prevalence (Positive Number/Total)
*bla*_TEM_	67.4% (31/46)
*bla*_SHV_	0.0% (0/46)
*bla*_CTX-MU_	60.9% (28/46)
*bla*_CTX-M9_	52.2% (24/46)
*bla*_OXA-1_	8.7% (4/46)
*armA*	0.0% (0/46)
*rmtA*	6.5% (3/46)
*rmtB*	4.3% (2/46)
*aac(6’)-1b*	15.2% (7/46)
*aac(3)-II*	21.7% (10/46)
*tetA*	95.7% (44/46)
*tetB*	2.2% (1/46)
*tetM*	58.7% (27/46)
*qnrA*	2.2% (1/46)
*qnrB*	32.6% (15/46)
*floR*	89.1% (41/46)
*mcr-1*	21.7% (10/46)
*oqxA*	76.1% (35/46)
*oqxB*	10.9% (5/46)
*sul1*	30.4% (14/46)
*sul2*	80.4% (37/46)
*fosA3*	19.6% (9/46)

**Table 6:** Changes in antimicrobia sensitivity of recipient bacteria and transconjugants.

Antimicrobial Agents	C600	EC027/T	EC035/T	EC038/T	EC027	EC035	EC038
penicillin	8	>512	>512	32	512	256	512
ceftazidime	1.25	10	1.25	1.25	80	1.25	1.25
streptomycin	16	256	128	128	>512	128	512
amikacin	8	128	16	4	>512	4	4
tetracycline	4	256	128	128	256	256	256
ciprofloxacin	<0.25	64	<0.25	<0.25	128	32	32
chloramphenicol	32	128	128	256	512	256	512

**Table 7:** Gene detection of conjugation resistance.

Isolates	Positive Resistance Genes
EC027/T	*bla*_OXA-1_ *, sul3, tetM, floR, aac(6′)-Ib, sul2, sul1*
EC035/T	*bla*_TEM_ *, sul3, tetA*
EC038/T	*bla*_TEM_ *, sul3, tetA*

## Data Availability

The others datasets used and/or analyzed during the current study are available from the corresponding author on reasonable request.

## Supplementary Materials

The following supporting information can be downloaded at: https://www.mdpi.com/article/10.3390/ani12080976/s1, Table S1: animals-12-00976-supplementary.xlsx.

## Abbreviations

*sul*: sulfonamide resistance genes; *E. coli: Escherichia coli*; ARG: Antimicrobial resistance genes; AMR: Antimicrobial resistance; DHPS: dihydro-pteroate synthase; ERIC-PCR: Enterobacterial Repetitive Intergenic Consensus—Polymerase chain reaction; PCR: Polymerase chain reaction; OD_600_: 600 optical density; MIC: Minimum Inhibitory Concentration; CLSI: Clinical and Laboratory Standards Institute; NCBI: National Center for Biotechnology Information.
